# Supplementary material for: Changes in early abortion access among out-of-state abortion patients in Illinois, following public insurance coverage through state Medicaid: A brief research report
Source: PLOS Glob Public Health. 2025 Nov 21;5(11):e0005432. doi: 10.1371/journal.pgph.0005432 (PMC12637953; doi:10.1371/journal.pgph.0005432)
Supplement: Attachment — Submitted filename: ResponseToReviewer_5.9.25.docx [file pgph.0005432.s002.docx]

Review Comments to the Author

Please use the space provided to explain your answers to the questions above. You may also include additional comments for the author, including concerns about dual publication, research ethics, or publication ethics. (Please upload your review as an attachment if it exceeds 20,000 characters)

Reviewer #1: The data used in this analysis have the potential to provide useful insights into changes in characteristics of people traveling to IL for abortion care, but I don’t think they are appropriate for the research question undertaken by the authors. To understand how changes in Medicaid funding might have spillover effects, you would need some kind of data that speak to (changes in) how patients from out of state paid for this care—ideally from the patient records themselves. Data from changes in funding streams/allocations from abortion funds could also be useful.

**Thank you for your feedback. We agree it would be helpful to have data on how out-of-state patients paid for their abortion and/or data from abortions funds for our research questions, but that information was not available in the dataset. Therefore, we refined our research question to assess if presenting early for an abortion (<= 11 weeks gestation) was associated with implementation of HB40 (2017 vs 2018-2019). We note in the Discussion that future research should explore how abortion funding in Illinois has changed since HB40. (lines 224-227)**

Similarly, national trends show increases in medication abortion and abortion at earlier gestations. It’s not appropriate to attribute (even if tentatively) this to changes in funding. I could even pose the counterargument that, given that IL has always been a “haven” for abortions in the second and later trimester, the decline in these procedures among residents from other states is a potentially negative development. In reality, I think other dynamics are at work and the authors are over-interpreting their findings.

**We appreciate your comment. Though national trends show increases in medication abortion and abortion at earlier gestations, it is unknown if these trends hold true for out-of-state patients. Further, there may be variation by state, particularly if a policy expanding abortion access – such as HB40 – were implemented. Though no longer the focus of our paper, we highlight in our discussion the possibility that changes in funding due to Medicaid coverage of abortion could have contributed to these shifts but that more research is needed: “Conversations with at least one abortion fund suggest that Medicaid coverage of abortion in Illinois resulted in more available funding for people in need of financial assistance who did not have or could not use Medicaid. However, more research is needed to understand if there was a significant reallocation of funds to out-of-state patients and if such funding contributed to earlier abortion access.” (lines 192-196). We also highlight in the Discussion section additional dynamics that could be contributing to earlier gestational age at the time of abortion, such as the opening of new clinics, expanded provision of abortion at existing clinics, and increased clinic staffing. (lines 177-180)**

Abortion funding is not well understood even by many people in the US and needs to be better explained. For this study it would be useful to know (1) if all the clinics in the study worked with or had abortion funds (2) the scale of this funding and (3) if/how these dynamics varied across the clinics.

**Thank you for your feedback. Unfortunately, our dataset had both limited and inconsistent data on abortion funding. As such, we are unable to assess the role, if any, that abortion funds played.**

Finally, access to abortion care, and changes in it, in the neighboring states are not adequately addressed—and I’m not sure they could be. Did the increase in patients from MO crowd out availability for people from IN? Did the “missing” travelers go to another IL clinic that is not in the study? Did residents in IN and WI have better access to care in 2019 than 2017 and, in turn, have less need to travel to IL?

**Thank you for your comment. While we agree that we cannot address how abortion-related policies in one bordering state impacted abortion access for patients coming from a different bordering state, we have added information on state policies that may be relevant to our findings on out-of-state abortion access in Illinois. For example, we found an increase in Missourians receiving care in Illinois and note in the Discussion section that this may have been driven by two Missouri policies that occurred during the study period: “The first occurred in October 2018, when new state requirements for abortion providers led to Missouri having only one abortion clinic. The following year, Missouri passed a law banning abortions after 8 weeks. It is possible that some of the overall increase in volume of out-of-state patients in our dataset can be attributed to Missouri state policies, making it difficult to assess the impact of HB40 on Missourians and other out-of-state patients.”** **(lines 200-205)**

More generally, I’m not sure the article is written for a global audience, it assumes a lot of “insider” knowledge of Medicaid, US abortion laws and geography. (Notably, these shortcomings can be remedied easily.)

**Thank you for your comment. We have revised our introduction to describe what Medicaid is, how it is funded, and summarize research on the impact of Medicaid coverage on abortion access: “Illinois is one of two Midwestern states where abortion services are covered by Medicaid [7,8], a public health insurance program for people earning low incomes. Though jointly funded by federal and state governments, Medicaid is mostly managed by states, resulting in state-level variation in eligibility requirements and services covered. Residents are generally unable to use Medicaid to cover the cost of health care services provided in other states. In Illinois, Medicaid coverage of abortion is associated with decreased out-of-pocket costs[9,10] and an increase in abortion volume [11], suggesting increased abortion access for Medicaid recipients..” (lines 39-45). We also added more details on U.S abortion laws: “****The United States (U.S.) Supreme Court’s decision to end the constitutional right to abortion in the 2022 *Dobbs v Jackson Women’s Health* *Organization* case resulted in each U.S. state enacting their own laws on abortion. As of January 2025, thirteen states ban abortion in most cases and six more ban access between 6 and 12 weeks of gestation,[1] forcing residents in these states to travel out-of-state for care.** **(lines 26-30), as well as Illinois’s location and abortion law: “****Bordering six states, including three which have banned abortion,[4] Illinois affirms abortion care for those seeking the service from nearby states. Illinois is one of the few states in the Midwest region of the U.S. where abortion is recognized as a right under the state constitution [5] and where later abortions (abortions obtained at or after 21 weeks gestation) are legal.[5,6].”(lines 34-38)**

More specific feedback includes:

The abstract devotes too much text citing tenuous arguments (see next sentence) and needs to focus more on the data and findings. In contrast to Line, 6 the research I’m familiar with suggests that Medicaid/insurance coverage increases access to second trimester care, not that it leads to abortions at earlier gestations.

**Thank you for your comment. We have rewritten our abstract to describe our revised analysis and findings:**

**In 2018, Illinois implemented House Bill 40 (HB40) which required Medicaid (means-tested public insurance) coverage of abortion care for Illinois residents. Medicaid coverage of abortion increases financial accessibility, which oftentimes leads to earlier access to care for covered patients. Since 2018, there has been an increase in the number of abortion patients and in the proportion of patients using Medicaid to pay for care. However, the impact of these changes on out-of-state patients – who cannot use Medicaid for abortion coverage – is unknown. To explore if Medicaid coverage of abortion impacted abortion access for out-of-state patients, we analyzed de-identified records of abortion visits among non-Illinois residents presenting for abortion care across 16 Illinois health centers. We used logistic regression to assess if presenting early for an abortion (<= 11 weeks gestation) was associated with implementation of HB40 (2017 vs 2018-2019). Although out-of-state residents were more likely to present early if they received abortion care in the post-HB40 period than pre-HB40 (81% at <=11 weeks in 2018-2019 vs 78% in 2017), multivariable regression show that HB40 was not associated with early abortion access when controlling for other patient characteristics. Out-of-state patients had higher odds of presenting <=11 weeks of gestation during the study period if they were a resident of a state bordering Illinois (OR 2.0, 95% CI 1.63-2.45, p<0.001) or were aged 25 or older (OR 1.49, 95%CI 1.34-1.65; p<0.001). After HB40, total volume of abortions (among residents and non-residents) increased, and among out-of-state patients, there was an increase in the proportion of Missouri residents and the proportion of patients over the age of 24. Given that policy changes in Missouri may have masked the influence of HB40 on out-of-state patients, more research is needed to understand any indirect impacts Medicaid coverage of abortion may have on out-of-state patients**. **(lines 2-22)**

**In regard to research on Medicaid leading to abortions at earlier gestations, a 2022 study(**[**https://journals.lww.com/greenjournal/abstract/2022/10000/association_of_availability_of_state_medicaid.14.aspx**](https://journals.lww.com/greenjournal/abstract/2022/10000/association_of_availability_of_state_medicaid.14.aspx)**) comparing state Medicaid coverage of abortion and abortion access found that patients residing in states with Medicaid coverage for abortion had lower odds of having abortions at more than 10 weeks of gestation when in the first trimester, compared with patients residing in states without Medicaid coverage for abortion. We have added another recently published study showing that average gestational age among Medicaid patients decreased after HB40 was implemented in Illinois. (**[**https://pmc.ncbi.nlm.nih.gov/articles/PMC12010279/**](https://pmc.ncbi.nlm.nih.gov/articles/PMC12010279/)**).**

Intro: The authors need to be explicit when the research they are citing is specific to IL and not “global” or necessarily relevant to all states where Medicaid covers abortion care.

**Thank you for your feedback. We have edited the introduction to cite Illinois-specific research on Medicaid coverage and make clear that this research is specific to that state:**

**“****In Illinois, Medicaid coverage of abortion is associated with decreased out-of-pocket costs[9,10] and an increase in abortion volume [11], suggesting increased abortion access for Medicaid recipients. However, out-of-state abortion patients who cannot use Medicaid have also been contributing to increased patient volume in Illinois. Between 2017 and 2023, the number of out-of-state abortion patients increased by 373%, compared to a 37% increase among residents during this same period.[12] Large increases in abortion patient volume may result in patients having abortions at later gestational ages due to potentially longer wait times between scheduling and having an appointment. A study examining the impact of HB40 on abortion access at one medical center in Illinois found that gestational age at the time of abortion increased by a median of eight days [9] and another study evaluating the influence of HB40 on abortion access across 18 Illinois health centers found that the time between scheduling and having an abortion increased by about 3 days[13]. This study also found an overall increase in gestational age after HB40, though there was a decrease in gestational age among Medicaid patients in particular[13]. Though longer wait times can contribute to later abortion access for some patients, there is also limited evidence suggesting that Medicaid coverage may indirectly lead to earlier abortion access for patients without Medicaid in Illinois. One study exploring the perspectives of providers and community stakeholders on the impact of HB40 found that some participants thought the law allowed resources to be shifted from Medicaid patients to other low-income patients that do not have or cannot use their insurance.[14] This potential increase in the availability of funding for some out-of-state patients may reduce the time needed to gather funds for their abortion and travel expenses, and could contribute to earlier gestational ages at the time of abortion.” (lines 44-64)**

Methods. It is worth nothing that the ILHD undercounts abortions, and/or that not all abortions are reported to them compared to counts obtained by the Guttmacher Institute. This, in turn, means a potentially smaller share of abortions in the state are captured by the clinics in the study (assuming they all report to the HD).

**Thank you for pointing this out. We have edited the methods section to note that not all abortions are reported to the IDPH (line 81) and note in the Discussion that because of this, our estimate of the proportion of abortions in our sample may be overestimated (lines 216-218).**

I had to read the sentence spanning lines 62-66 several times to understand the differences in PI laws between the states. Consider revising to that this distinction/relative burden is clearer.

**Thank you for your comment. We have revised our analysis to look at early abortion access among young people (aged <25 years) and no longer focus on minors so have removed the sentences about parental involvement policies for minors. We describe our rationale in the methods section: “With limited resources and transportation options, it may take women of color longer to gather sufficient funds for abortion care and finalize travel logistics, which may result in later gestational ages at the time of abortion.** **Similarly, young people may also find it difficult to gather sufficient funds for abortion care and travel, so we created a binary variable for age (<25 years and >= 25 years) and added this as a covariate.”** **(lines 109-113)**

The sample is too large to take chisq seriously—everything tends to be statistically significant. Consider using some form of simple logistic regression instead.

**Thank you for your suggestion. We have reanalyzed the data using both bivariate and multivariable logistic regression.**

Results
The stats in the first paragraph need context. I would expect abortion incidence to increase for IL residents in response to the change in Medicaid coverage so it’s hard to interpret the change in % of patients. Table 1 suggests a 38% increase but readers shouldn’t have to do the calculations by hand.

**Thank you for your comment. We have edited our paragraph describing the change in volume to the following:** **“Between 2017 and 2019, total volume of abortion patients (both residents and out-of-state patients) across all 16 clinics in our sample increased by 28% (from 17,373 in 2017 to 22,318 in 2019). Across the 12 clinics providing abortions prior to HB40, there was a 15% increase in abortion patients. Looking at volume increase by state of residency across these 12 clinics (n=10,049) during the same time period, the volume of abortion visits made by Illinois residents increased by 11% while the volume of visits made by out-of-state patients increased by 33%. Changes in patient volume varied at each of the 12 health centers between 2017 and 2019, with five experiencing volume increase of over 10%, two experiencing a decrease of over 10%, and five seeing little or no change in volume (0-10% change) (Table 2).” (lines 140-148)**

I initially looked for information on gestation and abortion type in Table 1, it’s not clear that this information is in the Figure. I’m not sure why you chose to display this information graphically and not in the table. The information in graphic format is a bit redundant since the measures are dichotomous.

**Thank you for your comment. We have removed the figure based on your recommendation.**

Discussion
You should lead with something else, for example that after HB40 was implemented there was an increase in N of out of state patients among the clinics that participated in the study.
**We appreciate your comment. We have revised our discussion section to fit our new analysis and our first sentence is “Between 2017 and 2019, there was an overall increase in the total number of abortion visits yet we found a higher proportion of out-of-state patients in our sample presented earlier for their abortion in 2019 than in 2017.” (lines 175-177)**

A number of abortion providers, and the majority of abortions provided, in the state are not included in the analysis. This needs to be addressed in the discussion. The patterns you uncovered may not be applicable to all people traveling from out of state.

**Thank you for your comment. While our sample does not capture all abortions provided by all abortion providers during the study period, we note in the methods section that the abortions captured by our sample represent close to 60% of abortions provided in the state during this study period. That said, we agree that we should acknowledge this gap in our limitations section and the resulting impact (which is that our findings may not be applicable to all people traveling to the state for abortion) and have added this as a limitation: “Although our sample captures almost 60% of** **out-of-state** **abortions provided in the state, it does not capture all abortion providers so our results may not be applicable to all out-of-state patients. In addition, not all abortions are reported to the IDPH, so our estimate of the proportion of abortions in our sample may be overestimated.” (lines 215-218)**

The text in lines 155-157 goes in the Conclusions.

**Thank you for your comment. The sentence was removed due to our revised analysis.**

Conclusions: I generally consider the concluding section/paragraph a place for “loftier” policy implications. This one focuses on speculations and implications for future research. I recommend revisiting the content and organization of the last two sections (Discussion and Conclusions).

**Thank you for your feedback. We have revised the content and organization of the Discussion and Conclusion sections to better reflect our revised analysis. As we were unable to assess the impact of HB40 on out-of-state patients, we highlighted the implications of our findings for future research instead of for policy.**
