# Supplementary material for: Changes in early abortion access among out-of-state abortion patients in Illinois, following public insurance coverage through state Medicaid: A brief research report
Source: PLOS Glob Public Health. 2025 Nov 21;5(11):e0005432. doi: 10.1371/journal.pgph.0005432 (PMC12637953; doi:10.1371/journal.pgph.0005432)
Supplement: Attachment — Submitted filename: Second Response to Reviewer_7.24.25.docx [file pgph.0005432.s003.docx]

Reviewer #1: This manuscript is substantially improved though the extensive revisions have introduced some new issues. In turn, I have some suggestions to make it a better paper.
Relatively minor but: You might consider de-identifying the clinics. Hope could be referred to as a large, independent clinic and you could list out the PP’s as Clinic 1 (or A), etc. There’s no need to reveal so much identifying information to external audiences (e.g., the abortion caseload at each individual clinic).

**Thank you for this suggestion. We have de-identified the clinics throughout the paper.**

Abstract: “Impact” is too strong of a word for this analysis. You are looking to see if patterns in out-of-state folks suggest an impact. You are unable to assert causality.

**Thank you – we have replaced the word “impact” with “associated with changes in abortion access” throughout the abstract.**

Intro: the first paragraph doesn’t appropriately set up the study. The text focuses on Dobbs and the impact it has had on abortion incidence in IL, but the study is pre-Dobbs. Most of the information in the first paragraph should be moved to the Discussion.

**Thank you for this feedback. We have revised the introduction to focus on describing Illinois prior to the Dobbs decision and have moved some of the post-Dobbs information to the Discussion section.**

The sentence on line 47-50 is a bit misfocused given that this analysis only goes through 2019—especially since in line 51 you revert back to the post-HB40 period. It might be more appropriate to indicate the proportion coming from oos during the study period, then note that this has increased substantially since Dobbs, making it even more important to understand these dynamics. (But also meaning they have limited usefulness given the recent surge, something you could address in the Discussion.) Also, this paragraph is very long and you might consider additional ways to break it up.

**We appreciate this feedback. We have revised these sentences (now lines 31-33) to report the proportion of out-of-state abortions compared to resident abortions between 2017-2019 and note that this has increased every year since the study period.** **We have also condensed our summaries of findings from previous research in order to shorten the length of the paragraph. Our Discussion section now addresses the importance of future research on post-*Dobbs* trends in abortion access for those traveling to Illinois.**

Methods: Line 73 needs clarification, presumably you emailed them asking them to participate in the study?

**Thank you for noticing this sentence needs clarification. We have clarified that we emailed them about their interest in participating in the study: “We emailed all health centers in Illinois offering medication and/or procedural abortions and that provided more than 500 abortions annually** **to ask if they were interested in participating in the study.” (lines 54-56)**

Lines 77-78: Be clear that these newly providing and new facilities are not included in this study. You can then remove the sentence on lines 121-123.

**We have made the number of clinics used in our analysis clearer in the methods section (lines 109-115) and specify in our results the number of clinics included in our analysis (the 12 clinics without the newly providing and new facilities versus across all 16 clinics). These clinics were not included in our analysis comparing pre- and post-HB40 or in our multivariable regression analysis but were included when we reported overall patient volume, distribution, and characteristics over the study period.** **We performed multivariable regression analysis both with and without these four clinics and our overall results did not change. We have therefore kept the sentences describing the percentages of resident and out-of-state visits at these clinics.**

Line 82: you should provide one or more citations to back up this statement. Perhaps comparing CDC and Guttmacher figures for the same year? Readers might want a senses of how in/accurate the HD data are.

**Thank you for this suggestion. The CDC abortion surveillance has the same numbers as the IDPH (I believe the IDPH reports their numbers to them) so we added information about how IDPH numbers compared to Guttmacher’s figures for the years in our study:**

**Compared to annual abortion data collected from the Guttmacher Institute (which gathers abortion data from a variety of sources including state health departments),[10,11] IDPH annual data on the total number of abortions was slightly higher (between 4%-6% higher) for each year of the study. (lines 67-70)**

Section that starts in line 99: You need to explain which statistical methods you will be using.

**Thank you for this feedback. We have re-organized the methods section and added the headers “Outcomes and measures” (line 85) and “Statistical analysis” (line 91) to make it clear where our statistical methods are described.**

Results: It’s confusing that the table uses different age categories than the text; for the latter, please provide the summary of over age 24. Or have a grouping in the table that has both larger and more detailed age groups.

**Thank you for this suggestion. We have added two larger age groups to Table 1 to show how patients 24 years or younger compared to patients older than 24 years old.**

Findings: Lines 157-161: It’s unclear why the figures in the text for Hope and Near North differ from those in the table. If this is not a mistake, please explain.

**Table 2 shows changes in volume of out-of-state and resident abortion patients at each clinic between 2017-2019. In lines 157-161 (now lines 152-156), we highlight changes in the proportion of out-of-state and resident abortions in a given clinic relative to other clinics. (for example, clinic 1 served 4.5% of out-of-state patients in our sample in 2017 vs 3.2% in 2019). We have clarified this by adding an appendix table showing these changes, as well as by adding the following sentence to the beginning of the paragraph: “****In addition to looking at changes in patient volume, we also looked at shifts in the share of out-of-state and resident abortions provided by clinics to identify any changes in the distribution of patients across clinics (Appendix 1)**.**” These calculations are also mentioned in our methods section (lines 87-89): “We also report on changes in out-of-state and resident patient volume between 2017 and 2019 at each clinic, as well as changes in the share of out-of-state and resident abortion patients seen across clinics.”**

Line 163: It would be useful to have this information (<=11 weeks) in a Table.

Thank you for this suggestion. We have added a table showing our bivariate and multivariable regression model results.

| ***Table 3. Variables associated and not associated with receiving care at ≤11 weeks gestation among out-of-state patients*** | | |
| --- | --- | --- |
|  | **Odds ratio (95% CI)** | **p-value** |
| **Bivariate model** |  |  |
| Post HB-40 (2018-2019) | 1.16 (1.05-1.29) | 0.004 |
| **Multivariable model** |  |  |
| Post HB-40 (2018-2019) | 1.07 (0.96-1.19) | 0.23 |
| Resident of bordering state | 1.89 (1.55-2.30) | <0.001 |
| Older than 24 years | 1.50 (1.35-1.66) | <0.001 |
| Racial identity: white | 1.09 (0.98-1.20) | 0.12 |

Discussion: It’s inappropriate to lead with the slight increase in <=11 weeks among out of state patients given that you just told us it was qualified—that it disappears when you take other factors into account. In turn, you need to revise the rest of the sentence in the paragraph. The sentence/information starting on line 184 is hard to follow and this would also be better in the “shortcomings” section. Or in the paragraph that starts on line 198.

**We appreciate this feedback. We now lead with the sentence “****Our study did not find an association between Medicaid coverage of abortion and early abortion access for out-of-state patients, however, we found that the odds of presenting early for an abortion increased for residents of bordering states and patients over 24 years old” and discuss possible reasons that could explain this finding. In the results, we added data on the increased number of out-of-state patients receiving early abortion care (“Across the 12 clinics providing abortions prior to HB40, the number of out-of-state patients receiving an early abortion increased by 40%”; lines 160-161) and, in the discussion, highlight reasons for this despite the increase in total patient volume over time: “****The increased number of out-of-state patients receiving an abortion at early gestational ages despite increases in total patient volume may have been due to increased clinic staffing, expanded provision of abortion services by clinics in our sample, and/or the opening of additional health centers offering abortion services. In addition to the three clinics in our sample that began providing medication abortion and the one clinic that opened during the study period, another new health center not in our sample opened near the Illinois-Missouri border in mid-October of 2019[19]. Additional facilities and staffing likely made it easier to meet the increased demand for abortion care and may have allowed a higher proportion of patients to be seen at earlier gestational ages.” (lines 199-206)**

**We have removed the sentence/information that started on line 184 and decided not to include it in our limitations section as we already note that one shortcoming of the study was not having data from all abortion providers in the state.**

Line 195: provide a citation.

**Thank you for this suggestion. We have decided to remove this sentence.**

Paragraph starting on line 207: You might also consider citing Chiu et al.: [https://www.mdpi.com/1660-4601/21/4/477](https://nam11.safelinks.protection.outlook.com/?url=https%3A%2F%2Furldefense.com%2Fv3%2F__https%3A%2F%2Fwww.mdpi.com%2F1660-4601%2F21%2F4%2F477__%3B!!MyIu0v6UfBA57LoN!-KFoztsTetS80-L4rtwCfxSGMZM2NNpBRXRXW0SIhqwNiwPFYfnT3cpYxzfAnnRT47LFx6sMUnF18wTASNwJ0fI%24&data=05%7C02%7Cczuniga%40ibisreproductivehealth.org%7Cf8394e4b2fd44d6a46f508ddb268a8b4%7C0bcca8c076334af4adfac4572a994f60%7C0%7C0%7C638862884757454784%7CUnknown%7CTWFpbGZsb3d8eyJFbXB0eU1hcGkiOnRydWUsIlYiOiIwLjAuMDAwMCIsIlAiOiJXaW4zMiIsIkFOIjoiTWFpbCIsIldUIjoyfQ%3D%3D%7C0%7C%7C%7C&sdata=RLOthWoGVl%2FQs1jSHcpFsUg5yGzWGhF8T%2FhUGr6bN1Y%3D&reserved=0)

**Thank you for recommending this citation. We have included Chiu et al.’s relevant findings into this paragraph: “Research has shown that young patients encounter unique logistical and privacy barriers to accessing abortion care, with one study finding that two-thirds of the 633 adolescents surveyed reported that someone had driven them to an abortion facility” (lines 185-188)**

Conclusions: this section doesn’t really align with the study findings, it’s largely focused on how much we don’t know about the current situation. You should use this section to better contextualize the findings in the current context.

**Thank you for this suggestion. We have revised the conclusion to align more with our study findings and have contextualized these findings in the post-Dobbs environment:**

**“****Although we did not find an association between Medicaid coverage of abortion for Illinois residents and earlier abortion access for out-of-state patients, our findings highlight that out-of-state patients are more likely to receive early abortion care if they reside in a border state and if they are older than 24 years. Future research should assess the factors that facilitate abortion access for out-of-state patients, especially adolescents and young adults. Such research is especially critical after the 2022 *Dobbs v Jackson Women’s Health Organization* decision, which has resulted in an increasing number of out-of-state abortion patients. Though our study suggests increased volume of patients did not translate to later abortion access for out-of-state patients, it remains unclear how very large increases in out-of-state patients over time impacted abortion care in Illinois and other states, and how clinics and abortion funds have handled the increase in demand. Finally, more studies are needed that assess direct and indirect impacts of supportive abortion policies, such as Medicaid coverage of abortion, on abortion access for those residing in and out of state.” (lines 234-245)**
