# Supplementary material for: Changes in early abortion access among out-of-state abortion patients in Illinois, following public insurance coverage through state Medicaid: A brief research report
Source: PLOS Glob Public Health. 2025 Nov 21;5(11):e0005432. doi: 10.1371/journal.pgph.0005432 (PMC12637953; doi:10.1371/journal.pgph.0005432)
Supplement: Attachment — Submitted filename: Response to Reviewer_Final.docx [file pgph.0005432.s004.docx]

Reviewer #1: I appreciate the authors’ incorporation of my feedback. This paper is much improved, and I only have a few suggestions to make it better—and one correction that needs to be made.

Abstract: You might be explicit about how/why Medicaid coverage for IL residents would make abortion more accessible for out-of-state patients. You could potentially remove the 2nd or 3rd sentence and explain that the ability of residents to use Medicaid might have freed up financial assistance for non-residents.

**Thank you for your comment. We have removed the third sentence as suggested and replaced it with the following sentence: “The ability of residents to use Medicaid may have increased the availability of financial assistance for non-residents.”**

Line. 69: The comparison of the IL HD and Guttmacher data is incorrect. While figures are not provided in the text, I see that Guttmacher reported 42080 and 52220 abortions in 2017 and 2019: [https://onlinelibrary.wiley.com/doi/epdf/10.1363/psrh.12215](https://nam11.safelinks.protection.outlook.com/?url=https%3A%2F%2Furldefense.com%2Fv3%2F__https%3A%2Fonlinelibrary.wiley.com%2Fdoi%2Fepdf%2F10.1363%2Fpsrh.12215__%3B!!MyIu0v6UfBA57LoN!_gpppsSlAUkvhsTpuZ25NsJWYH_Hep9UMzCe5XOYrA4ZA5a-RMW_YtLlWYM0_BlIzpqd8V53DILf8GiYtM0tg8M%24&data=05%7C02%7Cczuniga%40ibisreproductivehealth.org%7C327ef1f50a13454f5fde08de0a0ffacc%7C0bcca8c076334af4adfac4572a994f60%7C0%7C0%7C638959260937386984%7CUnknown%7CTWFpbGZsb3d8eyJFbXB0eU1hcGkiOnRydWUsIlYiOiIwLjAuMDAwMCIsIlAiOiJXaW4zMiIsIkFOIjoiTWFpbCIsIldUIjoyfQ%3D%3D%7C0%7C%7C%7C&sdata=SN%2BA4TtbOrnU%2FN%2BnShOI%2B6GPCDE%2BjEbAp0GuZlhno7w%3D&reserved=0).
By contrast, 39329 and 46517 abortions were reported to/by the IL HD (at least based on their published statistics): [https://dph.illinois.gov/data-statistics/vital-statistics/abortion-statistics](https://nam11.safelinks.protection.outlook.com/?url=https%3A%2F%2Furldefense.com%2Fv3%2F__https%3A%2Fdph.illinois.gov%2Fdata-statistics%2Fvital-statistics%2Fabortion-statistics__%3B!!MyIu0v6UfBA57LoN!_gpppsSlAUkvhsTpuZ25NsJWYH_Hep9UMzCe5XOYrA4ZA5a-RMW_YtLlWYM0_BlIzpqd8V53DILf8GiYBAuzs0c%24&data=05%7C02%7Cczuniga%40ibisreproductivehealth.org%7C327ef1f50a13454f5fde08de0a0ffacc%7C0bcca8c076334af4adfac4572a994f60%7C0%7C0%7C638959260937398561%7CUnknown%7CTWFpbGZsb3d8eyJFbXB0eU1hcGkiOnRydWUsIlYiOiIwLjAuMDAwMCIsIlAiOiJXaW4zMiIsIkFOIjoiTWFpbCIsIldUIjoyfQ%3D%3D%7C0%7C%7C%7C&sdata=0pne8c5QGIXglwTjPlnkPdLm479uLYzNOoLU7DZSgdE%3D&reserved=0)
These differences demonstrate that the HD stats are less complete and that this incompleteness may have increased/varied over time. Please revise the text accordingly. I suspect citation 11 is abortion by state of residence and the above PSRH citation is the more appropriate one.

**Thank you for your comment. We have replaced the citation with the report you suggested and have corrected the sentence to say that abortion numbers reported by the IDPH are lower than what is reported from Guttmacher:** **“Compared to annual abortion data collected from the Guttmacher Institute (which gathers abortion data from a variety of sources, including state health departments),[10] IDPH annual data on the total number of abortions was 6.5% and 11% lower for 2017 and 2019, respectively [6].”**

Line 154: It might be useful to indicate *where Clinic 8 was located in the state in case it can provide insights into the increase (e.g., near a state border?).

**Thank you for your comment. Clinic 8 is located in the Chicagoland area next to Lake Michigan, as are several other clinics that did not experience any changes in the share of out-of-state abortions provided, so location is likely not a factor for the changes we report. It is unclear what factors accounted for this change, so we have decided not to include any additional information about Clinic 8.**

Lines 159-163: not sure if you have the space, but it might be useful to have a table with this information. I had to read it a few times to make sure I understood what it was conveying.

**Thank you for this suggestion. To increase readability, we have decided to remove the first sentence of this paragraph to focus on our findings among the 12 clinics with pre- and post- HB40 data.**

Line 177: this is a run-on sentence.

**Thank you for noticing this. We have revised this sentence so that it is no longer a run-on sentence: “****Although our study did not find an association between Medicaid coverage of abortion and early abortion access for out-of-state patients, we found that the odds of presenting early for an abortion increased for residents of bordering states and patients over 24 years old.”**

Lines 185-191: These sentences could be condensed combined to make the text more efficient. I don’t know that you need to convey details like sample size in this context—you aren’t validating their findings, you’re just saying they suggest potential reasons for the associations you found. Ditto for lines 202-204, you don’t necessarily need to re-provide this detailed information; the point is that new clinics opened, including one that is not represented in the study.

**We appreciate your comment. We have removed details about the studies we refer to and have condensed this information to the following two sentences: “Research has shown that young patients encounter unique information and logistical barriers to accessing abortion care [16], and that many traveling out-of-state may not want to disclose their location or abortion intentions to family and friends. [17] Though disclosing one’s abortion plan to friends and/or family could result in financial and logistical support [18], young people unwilling to do so may have to delay care to gather funds for lodging and transportation costs in addition to funds for abortion care.**

**We have also removed the sentence with details on the new clinics and/or expanded provision of abortion services (originally lines 202-204)**

Line 207-208: please revise so that it reads more clearly. Is 33% vs 11% the share of out of state patients in the two time periods in your data or the difference between your and IDPH data (or something else)?

**Thank you for your comment. We have revised this sentence: “Similar to the data recorded by IDPH, our data also revealed a larger percentage increase in out-of-state abortion visits (33%) than visits by Illinois residents (11%) between 2017 and 2019.”**

Line 214: revise to read: indirect impact.

**Thank you. We have made this edit.**

Lines 221-223: considering revising to make it clear that there is still substantially more travel (across the US) for abortion compared to pre-Dobbs.

**Thank you for your comment. We have clarified that overall out-of-state travel for abortion is higher across the US after the Dobbs decision: “****Since the decision, out-of-state travel has varied nationwide, with increases in the first years following the decision and slight decreases in the latter following a surge in telehealth provision.[23] However, overall out-of-state travel is substantially higher across the country since the decision [24], and Illinois has continued to experience a large increase in of out-of-state abortion patients, with 39% of all abortions provided to out-of-state patients in 2024.[23]**
